# Methicillin-Resistant *Staphylococcus aureus*: Docking-Based Virtual Screening and Molecular Dynamics Simulations to Identify Potential Penicillin-Binding Protein 2a Inhibitors from Natural Flavonoids

**DOI:** 10.1155/2022/9130700

**Published:** 2022-05-04

**Authors:** Motahareh Masumi, Fatemeh Noormohammadi, Fatemeh Kianisaba, Fatemeh Nouri, Mohammad Taheri, Amir Taherkhani

**Affiliations:** ^1^Students Research Committee, Hamadan University of Medical Sciences, Hamadan, Iran; ^2^Department of Pharmaceutical Biotechnology, School of Pharmacy, Hamadan University of Medical Sciences, Hamadan, Iran; ^3^Department of Medical Microbiology, Faculty of Medicine, Hamadan University of Medical Sciences, Hamadan, Iran; ^4^Research Center for Molecular Medicine, Hamadan University of Medical Sciences, Hamadan, Iran

## Abstract

*Staphylococcus aureus* (*S. aureus*) is responsible for several disorders including skin and soft tissue infections, bacteremia, pulmonary infections, septic arthritis, osteomyelitis, meningitis, gastroenteritis, toxic-shock syndrome, and urinary tract infections. Methicillin-resistant *S. aureus* (MRSA) contains penicillin-binding protein 2a (SauPBP2a) responsible for catalyzing the peptidoglycan production within the bacterial cell wall. The binding affinity of SauPBP2a to beta-lactam antibiotics is low, and thus, it is necessary to discover new effective SauPBP2a inhibitors to combat mortality and morbidity in patients affected by MRSA. The binding affinity of 46 natural flavonoids to the SauPBP2a active site was examined via molecular docking analysis. The stability of docked poses associated with the top-ranked flavonoids was tested by performing molecular dynamics (MD) in 10 nanoseconds (ns) computer simulations. Kaempferol 3-rutinoside-7-sophoroside and rutin demonstrated a considerable binding affinity to the SauPBP2a active site (Δ*G*_binding_ < −11 kcal/mol). Their docked poses were found to be stable for 10 ns MD simulations. Kaempferol 3-rutinoside-7-sophoroside and rutin also exhibited salient binding affinity to the enzyme's allosteric site. This study suggests that kaempferol 3-rutinoside-7-sophoroside and rutin may be considered as drug candidates for therapeutic aims in several human infections associated with MRSA. Nevertheless, in vitro and in vivo confirmations are warranted.

## 1. Introduction

*Staphylococcus aureus* (*S. aureus*), a Gram-positive bacteria, is the leading cause of severe invasive and toxin-mediated disorders including skin and soft tissue infections, bacteremia, pulmonary infections, septic arthritis, osteomyelitis, meningitis, gastroenteritis, toxic-shock syndrome, and urinary tract infections. Methicillin-resistant *Staphylococcus aureus* (MRSA) refers to any strains of *S. aureus* that carry the *mec*A gene encoding penicillin-binding protein 2a (PBP2a), responsible for catalyzing the peptidoglycan production within the bacterial cell wall. As PBP2a has a lower binding affinity to beta-lactam-containing antibiotics compared to other PBPs, MRSA continues to catalyze bacterial cell wall synthesis in the presence of penicillin-derived antibiotics such as methicillin, oxacillin, nafcillin, and cephalosporins [1–7]. Thus, PBP2a in *S. aureus* (SauPBP2a) has been considered a potential target for developing specific inhibitors to combat MRSA, which will be accelerated by analyzing the three-dimensional structure of the SauPBP2a active site [8]. Furthermore, the allosteric site of SauPBP2a has also been considered for negative regulation of the enzyme's activity [9].

Organic compounds with antibacterial properties have sparked public interest in recent years for treating infections [10]. Flavonoids are one of the most important active compounds that naturally occur in photosynthesizing plants with several valuable properties in humans [11, 12]. Fruits and vegetables containing flavonoids have been widely used to treat human disorders [13]. Several flavonoids have been shown to possess antibacterial properties in previous research. In this regard, chrysin has been demonstrated to have bacteriostatic activity against *Escherichia coli* (*E. coli*) and *Pseudomonas aeruginosa* (*P. aeruginosa*).

Furthermore, baicalein has an inhibitory effect on the growth of *S. aureus* and *Bacillus subtilis*. Also, vitexin, saponarin, lucenin, apigenin, and luteolin have revealed bactericidal effects against *E. coli*, *Proteus mirabilis*, *Enterobacter cloacae*, *Klebsiella pneumoniae*, *Proteus vulgaris*, and *P. aeruginosa* [14]. In addition, kaempferol, myricetin, datiscetin, luteolin, and quercetin are effective against MRSA [15]. As a result, flavonoids have emerged as a primary research focus in the field of antibacterial medication discovery [13].

In the present study, we hypothesized that inhibition of PBP2a is a possible mechanism flavonoids use to combat MRSA growth. Thus, a docking-based virtual screening study was executed to examine the binding affinity of several natural flavonoids to the SauPBP2a active site. Molecular dynamics (MD) simulations were conducted to evaluate the stability of docked poses of top-ranked SauPBP2a active site inhibitors. The binding affinity of salient SauPBP2a active site inhibitors to the enzyme's allosteric site was also tested.

## 2. Materials and Methods

### 2.1. SauPBP2a and Preparation of Flavonoids' Structures

The three-dimensional coordinate of SauPBP2a was obtained from the Protein Data Bank (PDB) database, which is available at https://www.rcsb.org. Two PDB codes including 1MWT (X-ray resolution = 2.45 Å [8, 16]) and 4CJN (X-ray resolution = 1.95 Å [17]) were selected for docking analyses with the active and allosteric sites of the enzyme, respectively. The 1MWT and 4CJN files included two polypeptide chains as A and B. Chain B was selected for further analysis, which contained 646 and 642 residues in the 1MWT and 4CJN files, respectively. The chain B also included penicillin G (PDB ID: PNM) and (E)-3-(2-(4-cyanostyryl)-4-oxoquinazolin-3(4H)-yl) benzoic acid (PDB ID: QNZ) in the 1MWT and 4CJN PDB files, respectively; these ligands were removed from the SauPBP2a structure before molecular docking and dynamics studies.

Energy minimizing (EM) of SauPBP2a with two different codes was carried out before docking operations using the Swiss-PDB Viewer version 4.1.0, which is available at https://spdbv.unil.ch/ [18]. A total of 46 natural flavonoids were docked with the active site of SauPBP2a to identify potential inhibitors of the enzyme's catalytic domain. Next, molecular docking analysis was done using the known PBP inhibitors, including penicillin G and methicillin. Due to the low binding affinity of SauPBP2a to the beta-lactam-containing antibiotics, penicillin G and methicillin were considered control negative in this study. Furthermore, previous studies have reported that “oxadiazole” [19] and “ceftobiprole” [20] had exhibited high binding affinity to the SauPBP2a active site and hence were considered control positive in our work.

The two-dimensional (2D) coordinates of flavonoids, methicillin, and ceftobiprole were principally collected from the PubChem database (https://pubchem.ncbi.nlm.nih.gov) as a structure data file (SDF). Subsequently, the Cactus web server was used to achieve PDB formats from the SDF files, available at https://cactus.nci.nih.gov/translate/. The three-dimensional structure of penicillin G was collected from the 1MWT file, while the construction of oxadiazole was drawn by the ChemDraw software version 12.0.2.1076. The EM was administered for all small molecules using the HyperChem software version 8.0.10 [21].

### 2.2. Molecular Docking with the SauPBP2a Active Site

A windows-based program system was used with the following features: system type, 64 bit; processor, Intel Core i7; installed memory, 32 GB. Docking-based virtual screening was executed using AutoDock 4.0 (https://autodock.scripps.edu) [22]. The AutoDock software applies limited flexibility in the receptor and uses the Lamarckian genetic algorithm to predict the docked pose of the ligand [23]. The SauPBP2a catalytic site was considered the receptor for executing docking analyses with flavonoids, control negative, and control positive compounds. After analyzing the interactions between nitrocefin, penicillin G, methicillin, and the residues within the SauPBP2a catalytic domain from the study by Lim and Strynadka [8], a total of 14 main residues were identified inside the PBP2a active site including Ser337, Lys340, Ser403, Lys406, Tyr446, Ser462, Asn464, Thr500, Ser548, Gly549, Ser598, Gly599, Thr600, and Met681. Thus, these amino acids were considered as a receptor site for docking analysis to identify potential inhibitors of the enzyme's active site. The grid box settings were as follows: X-dimension, 60; Y-dimension, 66; Z-dimension, 60; X-center, −34.264; Y-center, 44.65; Z-center, 66.778; spacing, 0.375 Å. The number of docked poses for each component was set at 50, where the docked model with the lowest ∆*G*_binding_ involved in the largest cluster along with the largest number of models using a root mean square deviation (RMSD), tolerance of 2.0 Å, was considered as a binding affinity of the ligand to the receptor site.

### 2.3. Cross-Validation Study

Cross-validation of docking studies was executed for top-ranked SauPBP2a active site inhibitors based on the Δ*G*_binding_ values achieved from the AutoDock tool (Δ*G*_binding_ < −10 kcal/mol). This was performed using the Schrödinger Maestro docking software version 10.2 [24, 25]. In this regard, docking scores were calculated, and the prime MM-GBSA method was used to determine the relative binding energies of ligands. The Glide docking system was employed to evaluate the binding affinity of compounds to the SauPBP2a active site. The lowest Glide score (dock score) for each ligand was considered the best-docked model. More details regarding settings in the Schrödinger Maestro docking software have been reported in a previous study by Azadian et al. [26].

### 2.4. MD Simulations

To examine the stability of docked poses among top-ranked flavonoids and the SauPBP2a active sites, MD was simulated by applying the Discovery Studio Client software (version 16.1.0.15350). Only ligands that demonstrated considerable affinity to the enzyme's active site using the AutoDock tool and Schrödinger docking software were considered for MD analysis. The advanced settings for computer simulations were as follows: MD time: 10 nanoseconds (10 ns), solvation model: explicit periodic boundary; cell shape: orthorhombic; minimum distance from the boundary: 10 Å; solvent: water; target temperature: 310 K; force field: CHARMm; charge distribution: point. In addition, the time evolution of root mean square deviation (RMSD) of backbone atoms and root mean square fluctuation (RMSF) of SauPBP2a complexed with the best inhibitors was also analyzed. The BIOVIA Discovery Studio Visualizer version 19.1.0.18287 was then utilized to demonstrate the interactions among top-ranked flavonoids and the residues inside the SauPBP2a catalytic site and to superimpose protein-ligand complexes before and after MD simulations.

### 2.5. Molecular Docking with the SauPBP2a Allosteric Site

This study considered the best SauPBP2a active site inhibitors to evaluate their binding affinity to the enzyme's allosteric site. The grid box options at this stage followed the settings of the study by Ibrahim et al. [9]: *X*-dimension: 52; *Y*-dimension: 52; *Z*-dimension: 52; *X*-center: 9.658; *Y*-center: −1.662; *Z*-center: −70.269; spacing: 0.375 Å.

## 3. Results

### 3.1. Molecular Docking with the SauPBP2a Active Site

**Figure 1:**
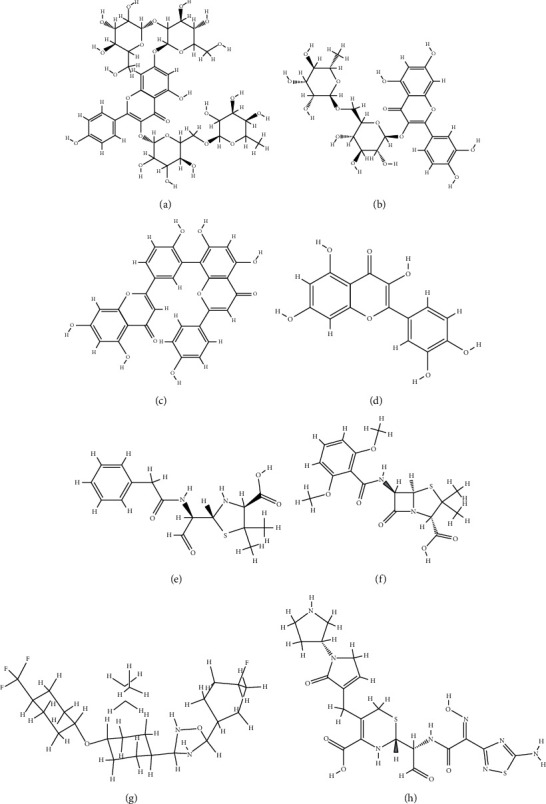
Two-dimensional structures of (a) kaempferol 3-rutinoside-7-sophoroside, (b) rutin, (c) amentoflavone, (d) quercetin, (e) penicillin G, (f) methicillin, (g) oxadiazole, and (h) ceftobiprole.

**Figure 2:**
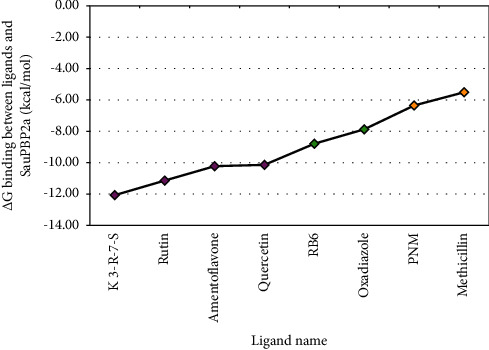
The estimated binding energy for top-ranked flavonoids and control compounds. *X*-axis: green and orange diamonds indicate control positive and control negative compounds, respectively. *Y*-axis represents the estimated binding energy (kcal/mol). K 3-R-7-S, kaempferol 3-rutinoside-7-sophoroside; RB6, ceftobiprole; PNM, penicillin G.

**Figure 3:**
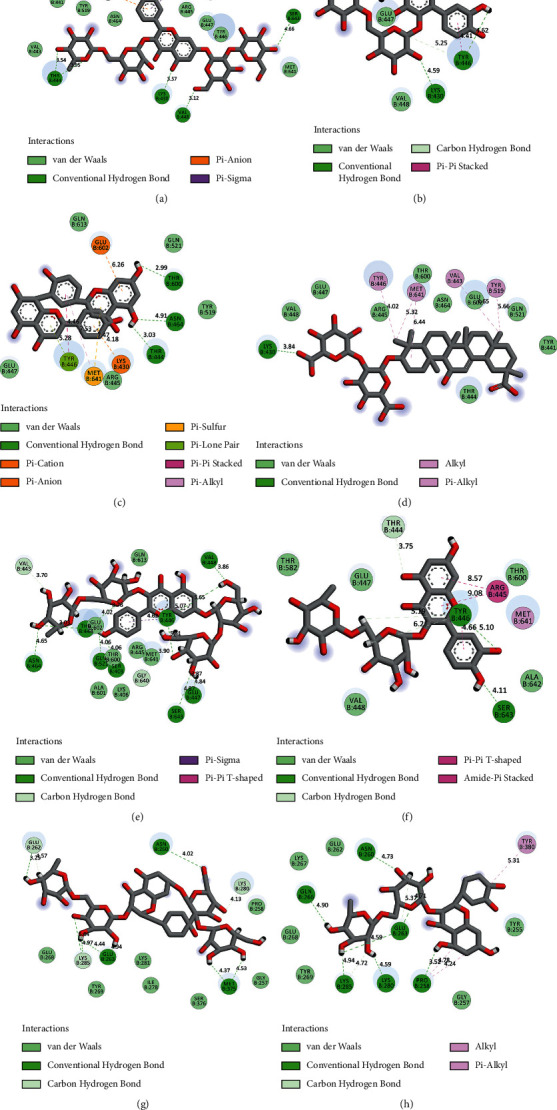
Interaction modes between residues within the SauPBP2a active site and top-ranked flavonoids. Before MD simulations using the AutoDock tool: (a) kaempferol 3-rutinoside-7-sophoroside, (b) rutin, (c) amentoflavone, and (d) quercetin. After 10 ns MD simulations: (e) kaempferol 3-rutinoside-7-sophoroside and (f) rutin. Using the Schrödinger Maestro docking software: (g) kaempferol 3-rutinoside-7-sophoroside and (h) rutin. SauPBP2a, *Staphylococcus aureus* penicillin-binding protein 2a.

**Figure 4:**
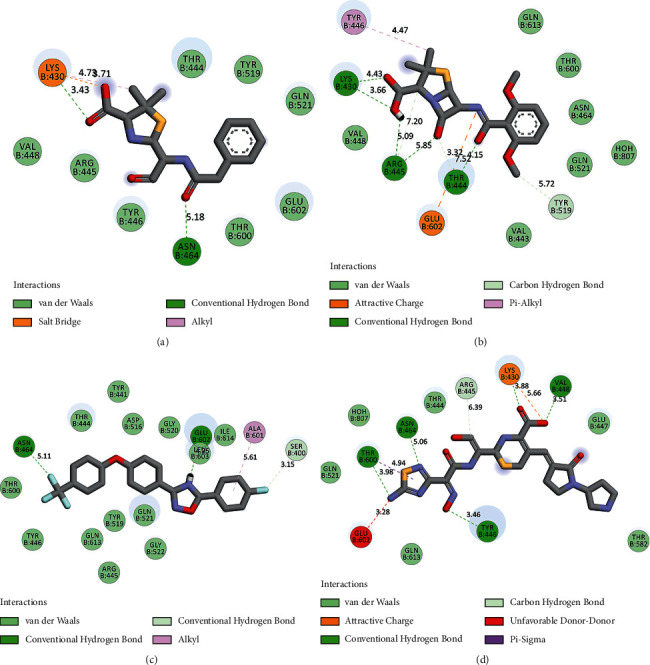
Interaction modes between residues inside the SauPBP2a active site and control positive and negative compounds. (a) Penicillin G, (b) methicillin, (c) oxadiazole, and (d) ceftobiprole. SauPBP2a, *Staphylococcus aureus* penicillin-binding protein 2a.

**Figure 5:**
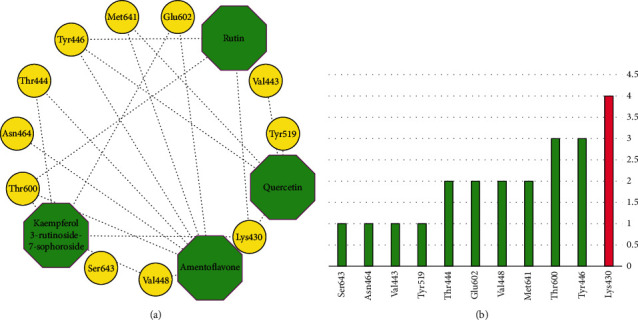
(a) A unique network demonstrating possible interactions between top-ranked flavonoids and the residues within the SauPBP2a active site. (b) Degree diagram. *X*-axis and *Y*-axis demonstrate the name of amino acid inside the SauPBP2a active site and its corresponding degree, respectively. SauPBP2a, *Staphylococcus aureus* penicillin-binding protein 2a.

It was found that 9 of the flavonoids can potentially bind to the SauPBP2a catalytic site on the nanomolar scale (nM). A total of 37 compounds revealed an inhibitory effect on SauPBP2a active site at the micromolar (uM) scale. Four components, including kaempferol 3-rutinoside-7-sophoroside, rutin, amentoflavone, and quercetin, bounded to the SauPBP2a active site with the salient affinity of ∆*G*_binding_ < −10 kcal/mol (Supplementary Table 1). Thus, these compounds found the top SauPBP2a active site inhibitors among the tested flavonoids based on the AutoDock tool and were considered for further analyses using the Schrödinger Maestro docking software. Ceftobiprole and oxadiazole exhibited a mild binding affinity to the enzyme's active site with the ∆*G*_binding_ values of −8.79 and −7.87 kcal/mol, respectively.  Figure 1 shows the two-dimensional structures of kaempferol 3-rutinoside-7-sophoroside, rutin, amentoflavone, quercetin, and control negative and positive compounds in this study, which were achieved by the ChemDraw Ultra version 12.0.2.1076. Of note, the ∆*G*_binding_ between penicillin G as well as methicillin and SauPBP2a active site was calculated to be −6.35 and −5.51 kcal/mol, respectively.  Figure 2 shows the ∆*G*_binding_ values between top-ranked flavonoids, control inhibitors, and the SauPBP2a active site. In comparison, the details of energies between top-ranked flavonoids, control inhibitors, and the enzyme's catalytic domain are given in Table 1. All hydrogen, hydrophobic, electrostatic, and miscellaneous interactions between kaempferol 3-rutinoside-7-sophoroside, rutin, amentoflavone, quercetin, and residues inside the SauPBP2a catalytic site were analyzed before and after MD simulations (Table 2 and Figure 3). In this regard, kaempferol 3-rutinoside-7-sophoroside revealed the most H-bonds before and after MD analyses. The H-bonds with a distance of >5 Å were not considered significant interactions. Furthermore, Figure 4 shows the interaction modes between control compounds and the residues inside the enzyme's active site. All possible interactions between kaempferol 3-rutinoside-7-sophoroside, rutin, amentoflavone, quercetin, and the residues within the SauPBP2a catalytic site are shown in Figure 5(a) as a single graph. This network was achieved using the Cytoscape software (3.8.0; https://www.cytoscape.org) [27]. Subsequently, the degree of each residue was calculated using the Cytoscape network analyzer tool, illustrating the number of interactions between each residue and the top-ranked flavonoids. Lys430 indicated the highest degree between the residues (Figure 5(b)).

### 3.2. Cross-Validation and MM-GBSA Analyses

Schrödinger Maestro docking scores were calculated for kaempferol 3-rutinoside-7-sophoroside, rutin, amentoflavone, and quercetin. Kaempferol 3-rutinoside-7-sophoroside revealed the highest binding affinity to the active site of SauPBP2a, followed by rutin with the dock scores of −12.697 and −9.341 kcal/mol, respectively (Table 3). Thus, these two compounds were considered salient inhibitors of the SauPBP2a active site and were selected for further MD analysis. Meanwhile, MD simulations were performed based on the complexes achieved from the AutoDock software. Furthermore, the interaction modes between kaempferol 3-rutinoside-7-sophoroside, rutin, and residues within the sauPBP2a active site were also studied based on the complexes achieved from the Schrödinger Maestro docking tool (Table 2, Figures 3(g) and 3(h)). The prime MM-GBSA analysis showed each compound's relative binding-free energy (Δ*G* bind), with the results given in Table 4. The expanded formula is given as follows [28]: Δ*G* (bind) = Δ*G* (solv) + Δ*E* (MM) + Δ*G* (SA).

### 3.3. MD Simulations

**Figure 6:**
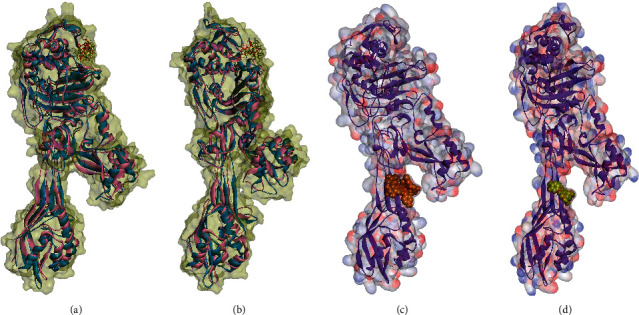
(a)-(b) Docking analyses with the active site of SauPBP2a: superimposed structures of SauPBP2a complexed with (a) kaempferol 3-rutinoside-7-sophoroside and (b) rutin before MD simulations (blue chains) and after MD simulations (pink chains). Red and yellow colors represent ligands before and after MD simulations, respectively. Docking models of SauPBP2a allosteric site with (c) kaempferol 3-rutinoside-7-sophoroside and (d) rutin in CPK mode.

**Figure 7:**
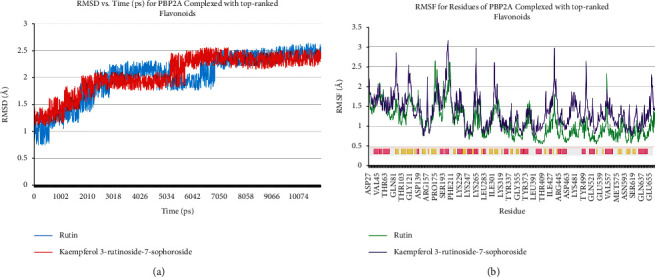
(a) Time evolution of RMSD of backbone atoms and (b) RMSF for SauPBP2a complexed with kaempferol 3-rutinoside-7-sophoroside and rutin. The secondary structure of the protein was achieved from the RCSB database. Pink and yellow colors illustrate helics and beta-strand structures, respectively. RMSD, root mean square deviations; RMSF, root mean square fluctuation.

MD simulation was done to assess the stability of docked poses of kaempferol 3-rutinoside-7-sophoroside and rutin.  Figure 6 shows the superimposed structures of SauPBP2a complexed with kaempferol 3-rutinoside-7-sophoroside and rutin before and after MD simulations. Furthermore, the RMSD of backbone atoms and RMSF for SauPBP2a complexed with kaempferol 3-rutinoside-7-sophoroside and rutin are shown in Figure 7. As shown in Figure 7, it seems that the helix structures are more stable than the other secondary structures in SauPBP2a.

### 3.4. Molecular Docking with the SauPBP2a Allosteric Site

The results showed that the potential inhibitors of SauPBP2a active site including kaempferol 3-rutinoside-7-sophoroside and rutin also had an excellent binding affinity to the allosteric site of the enzyme with the criteria of Δ*G*_binding_ values as −14.35 and −9.68 kcal/mol, respectively (Table 1).

## 4. Discussion

Antibiotic resistance has emerged in response to the overuse of currently available antibiotics [10]. Thus, it is necessary to discover and design novel antibacterial compounds with new formulations to overcome serious infections [29]. MRSA infection is one of the most frequent causes of hospital-acquired conditions and is currently associated with poor prognosis and increased mortality/morbidity [30]. In the present study, molecular docking and dynamics simulations were performed to identify potential inhibitors of the SauPBP2a active site. However, targeting the PBP2a allosteric site is another alternative strategy to reduce SauPBP2a activity. As such, we also evaluated the binding affinity of our top-ranked PBP2a active site inhibitors including kaempferol 3-rutinoside-7-sophoroside and rutin to the allosteric site of PBP2a. We used the same protein Ibrahim et al. [9] used in their study (PDB ID: 4CJN; chain B). Interestingly, kaempferol 3-rutinoside-7-sophoroside and rutin exhibited high binding affinity to the PBP2a allosteric site with the Δ*G*_binding_ values of −14.35 and −9.68 kcal/mol, respectively.

Kaempferol is a flavonoid compound with various health benefits such as anti-inflammatory, anticancer, liver-protective, antiobesity, antidiabetes, and heart-protective effects. It is found in fruits and vegetables, tea, beans, cabbage, broccoli, barriers, grapes, apples, and citrus fruits [31]. Previous research has shown that kaempferol and its many derivatives have antimicrobial properties. Qui et al. [32] demonstrated that two of the kaempferol derivatives named 3-O-[*β*-d-glucopyranosyl-(1 ⟶ 2)-*α*-l-rhamnopyranosyl-(1 ⟶ 6)]-*β*-d-glucopyranoside and kaempferol 3-O-[*β*-d-xylopyranosyl-(1 ⟶ 2)-*α*-l-rhamnopyranosyl-(1 ⟶ 6)]-*β*-d-glucopyranoside revealed considerable antibacterial actions against *S. aureus*, *E. coli*, *Salmonella enteriditis*, *Bacillus thuringiensis*, *Aspergillus niger*, and *Rhizopus nigricans*. These two compounds were extracted from *Camellia oleifera* using the continuous phase change extraction approach. Furthermore, Cruz et al. [10] demonstrated that the combination of kaempferol 7-O-*β*-D-(6″-O-cumaroyl)-glucopyranoside with the amikacin and gentamicin at a concentration of 128 *μ*g/mL led to synergistic actions against *S. aureus* and *E. coli* and significantly diminished the minimum inhibitory concentration (MIC) from 16 to 4 and 88 *μ*g/mL, respectively. One year later, Kannanoor et al. [33] reported that the silver nanoparticles (AgNPs) conjugated with kaempferol (K-AgNPs) demonstrated a considerable bactericidal activity against various bacterial strains including *E. coli*, *Bacillus subtilis*, and *S. aureus*. They used several experimental approaches, including live/dead bacterial ratio analysis, lactate dehydrogenase, and lipid peroxidation analyses. According to our results, it was estimated that kaempferol has a mild binding affinity to the catalytic site of SauPBP2a (∆*G*_binding_ =  −6.12 kcal/mol; *K*i = 32.86 *µ*M), while two of the kaempferol derivatives including kaempferol 3-rutinoside-7-sophoroside and kaempferol 3-rutinoside-4′-glucoside were found to bind to the SauPBP2a active site at the *K*i values of 1.42 nM and 562.45 nM, respectively. This suggests that the sugar moiety has enhanced the binding affinity of kaempferol to SauPBP2a active site. Using the AutoDock and Schrödinger Maestro docking tools, kaempferol 3-rutinoside-7-sophoroside revealed a high binding affinity to the SauPBPa2 active site with the ∆*G*_binding_ of −12.07 and −12.697 kcal/mol, respectively. Before MD, kaempferol 3-rutinoside-7-sophoroside indicated six hydrogen, one electrostatic, and one hydrophobic interaction with the Lys430, Thy444, Val448, Thr600, Glu602, and Ser643 inside the catalytic domain of SauPBPa2. It formed 12 hydrogen and 3 hydrophobic interactions with Ser403, Val443, Tyr444, Tyr446, Glu447, Val448, Asn464, Gln521, Gly640, and Ser643 within the SauPBP2a active site after 10 ns MD simulation.

Rutin is an organic flavonol with several pharmacological features such as antioxidant and antibacterial properties [34–37]. In a previous report, a combination of rutin and florfenicol exhibited enhanced antibacterial activity against multidrug-resistant (MDR) *Aeromonas hydrophila*, suggesting that the use of organic compounds in combination with traditional antibiotics may result in synergistic inhibition of the growth of MDR bacteria [29]. Recently, Rodríguez-Valdovinos and Salgado-Garciglia [38] designed a study to examine the antibacterial and antioxidant effects of *Verbesina sphaerocephala* (*V. sphaerocephala*) leaf and flower extracts, in which rutin is the main phenolic component. Rodríguez-Valdovinos and Salgado-Garciglia [38] reported that *V. sphaerocephala* extracts demonstrated significant antibacterial effects on *S. aureus* and *E. coli*. Our AutoDock results found that rutin can bind to the SauPBPa2 catalytic domain at the nanomolar concentration (*K*i = 436.10 nM) with an estimated Gibbs free energy of −11.14 kcal/mol. Furthermore, the Schrödinger Maestro docking software estimated that the dock score between rutin and SauPBP2a active site was −9.341 kcal/mol. Before MD simulations, rutin formed 3 hydrogen and 1 hydrophobic interaction with Lys430, Tyr446, and Thr600 within the SauPBPa2 active site. This flavonoid showed 2 hydrogen and 3 hydrophobic interactions with Tyr444, Arg445, and Ser643 inside the SauPBP2a active site after 10 ns MD simulation.

Amentoflavone is a bioflavonoid metabolite with miscellaneous beneficial properties including anti-inflammatory, neuroprotective, antiviral, and anticancer effects [39–42]. It has been widely purified from *Hypericum perforatum*, *Ginkgo biloba*, and *Selaginella tamariscina* [43]. Hwang et al. [44] performed a study to evaluate the antibacterial effects of amentoflavone alone, isolated from *Selaginella tamariscina*, and in combination with other antibiotics including chloramphenicol, cefotaxime, and ampicillin. The authors reported synergistic results of combined amentoflavone with other traditional antibiotics on various Gram-positive and Gram-negative bacteria including *S. aureus*, *E. coli*, *Enterococcus faecium*, and *Pseudomonas aeruginosa* (*P*. *aeruginosa*). This was carried out by applying the Clinical and Laboratory Standards Institute guideline [45]. The authors suggested that the enhanced production of hydroxyl radicals by amentoflavone is potentially the main reason for the synergistic outcome. Our results found that amentoflavone can bind to the SauPBPa2 active site at the nanomolar concentration (*K*i = 31.63 nM) and with an estimated free binding energy of −10.23 kcal/mol. Our docking results indicated that amentoflavone revealed 3 hydrogen, 4 hydrophobic, 2 electrostatic, and 2 miscellaneous interactions with Lys430, Thr444, Tyr446, Val448, Asn464, Thr600, Glu602, and Met641 within the SauPBPa2 active site.

Quercetin is predominantly found in vegetables and fruits such as apples, broccoli, berries, onions, kales, tee, and red grape. Quercetin has several valuable characteristics, including antioxidant, anti-inflammatory, anticarcinogenic, and antiviral properties. It also reduces the risk of infectious diseases and promotes mitochondrial biogenesis [46–51]. Wang et al. [52] performed in vivo and in vitro studies to examine the antibacterial effects of quercetin on cecal microbiota of Arbor Acre broiler chickens, as well as its mechanism of action. Wang et al. [52] demonstrated that quercetin significantly reduced the total amount of *S. aureus* (*P* < 0.01), *P*. *aeruginosa* (*P* < 0.05), *Salmonella enterica* serotype Typhimurium (*P* < 0.01), and *E. coli* (*P* < 0.01) compared to the negative control. Furthermore, the authors provided the in vitro clue that quercetin damages the membrane and cell walls of *S. aureus* and *E. coli*, indicating that it could be used as an alternative antibacterial feed for animals. Based on our findings, quercetin revealed 1 hydrogen and 6 hydrophobic interactions with Lys430, Val443, Tyr446, Tyr519, and Met641 within the PBPa2 active site.

The stability of docked poses of kaempferol 3-rutinoside-7-sophoroside and rutin was studied by performing MD in 10 ns computer simulations. It was suggested that the docked poses of these 2 flavonoids were stable in 10 ns simulation. In addition, kaempferol 3-rutinoside-7-sophoroside and rutin also revealed considerable binding affinity to the allosteric site of the enzyme. Thus, these compounds may be considered effective inhibitors of SauPBP2a, though this needs in vitro and in vivo confirmations in future studies.

The present study had a principal limitation. MD is a computer simulation approach analyzing the physical movements of atoms and molecules in an environment similar to reality. Thus, a supercomputer with a powerful processor and random access memory (RAM) is an indispensable tool for executing these types of time-consuming procedures. Unfortunately, the supercomputer was unavailable for our research team to run MD simulations for more significant periods. Each MD simulation (10 ns) took about one month with the computer we had. We strongly believe that simulating MD for a greater period of times results in more reliable results.

## 5. Conclusion

46 flavonoids were docked to the SauPBP2a active site using the AutoDock tool to estimate the binding affinity of the selected herbal flavonoids to the target protein. A cross-validation study was performed for compounds with the criteria of Δ*G*_binding_ < −10 kcal/mol. Next, MD simulation was executed for compounds that exhibited considerable binding affinity to the enzyme's active site using AutoDock and Schrödinger Maestro docking tools. The results suggest that kaempferol 3-rutinoside-7-sophoroside and rutin have a high affinity of binding to the SauPBP2a active site. Their docked poses were stable in the 10 ns simulation. They also exhibited excellent binding affinity to the enzyme's allosteric site. Thus, it may be speculated that kaempferol 3-rutinoside-7-sophoroside and rutin could be helpful in the therapeutic aims of MRSA infections. Nevertheless, further in vivo and in vitro inhibition experiments are warranted in the future.

## Figures and Tables

**Table 1:** Details of energies and inhibition constant values between top-ranked flavonoids, control compounds, and SauPBP2a achieved from the AutoDock tool.

(A) Docking results with the active site of the enzyme						
Ligand name	Final intermolecular energy (kcal/mol)	Final total internal energy (kcal/mol)	Torsional free energy (kcal/mol)	Unbound system's energy (kcal/mol)	Estimated free binding energy (kcal/mol)	*K*i
Kaempferol 3-rutinoside-7-sophoroside	−12.8	−3.4	2.7	−1.4	−12.1	1.4 nM
Rutin	−8.0	−10.2	4.8	−2.2	−11.1	6.8 nM
Amentoflavone	−9.7	−4.4	2.7	−1.2	−10.2	31.6 nM
Quercetin	−9.9	−6.6	4.5	−1.9	−10.1	36.6 nM
PNM (ctrl ‒)	−7.9	−1.1	1.8	−0.9	−6.4	22.1 uM
Methicillin (ctrl ‒)	−6.8	−1.8	1.8	−1.3	−5.5	91.2 uM
Oxadiazole (ctrl +)	−9.2	−0.9	1.5	−0.7	−7.9	1.7 uM
Ceftobiprole (ctrl +)	−10.6	−2.1	2.7	−1.2	−8.8	362.2 nM

(B) Docking results with the allosteric site of the enzyme						
Ligand name	Final intermolecular energy (kcal/mol)	Final total internal energy (kcal/mol)	Torsional free energy (kcal/mol)	Unbound system's energy (kcal/mol)	Estimated free binding energy (kcal/mol)	*K*i

Kaempferol 3-rutinoside-7-sophoroside	−7.3	−17.8	7.5	−3.3	−14.4	30.30 pM
Rutin	−7.4	−9.8	5.1	−2.4	−9.7	79.98 nM

SauPBP2a, *Staphylococcus aureus* penicillin-binding protein 2a; PNM, penicillin G; Ki, inhibition constant; nM, nanomolar; pM, picomolar.

**Table 2:** Interaction modes between top-ranked SauPBP2a inhibitors and active site of the enzyme.

(A) Molecular dockings performed using the AutoDock tool, and subsequently, the interactions between ligands and residues studied before MD simulations.				
Ligand name	Hydrogen bond (distance Å, subtype)	Hydrophobic interaction (distance Å, subtype)	Electrostatic (distance Å, subtype)	Miscellaneous (distance Å, subtype)
Kaempferol 3-rutinoside-7-sophoroside	Thr444 (3.54 classical, 3.95 classical); Glu602 (3.18 classical); Ser643 (4.66 classical); Val448 (3.12 classical); Lys430 (3.57 classical)	Thr600 (5.83 pi-alkyl)	Glu602 (6.55 pi-anion)	NA
Rutin	Thr600 (3.19 classical); Tyr446 (4.62 classical); Lys430 (4.59 classical)	Tyr446 (4.41 pi-pi stacked)	NA	NA
Amentoflavone	Thr600 (2.99 classical); Asn464 (4.91 classical); Thr444 (3.03 classical)	Tyr446 (4.46 pi-pi stacked); Val448 (5.97 pi-alkyl); Met641 (7.53 pi-alkyl); Lys430 (4.18 pi-alkyl)	Lys430 (4.18 pi-cation); Glu602 (6.26 pi-anion)	Tyr446 (5.28 lone pairs); Met641 (7.47 sulfur)
Quercetin	Lys430 (3.84 classical)	Tyr446 (4.02 pi-alkyl, 4.06 alkyl); Met641 (5.32 alkyl, 6.44 alkyl); Val443 (4.65 alkyl); Tyr519 (5.66 pi-alkyl)	NA	NA

(B) Molecular dockings executed using the AutoDock tool, and subsequently, the interactions between ligands and residues studied after 10 ns MD simulations.				
Ligand name	Hydrogen bond (distance Å, subtype)	Hydrophobic interaction (distance Å, subtype)	Electrostatic (distance Å, subtype)	Miscellaneous (distance Å, subtype)

Kaempferol 3-rutinoside-7-sophoroside	Val443 (3.70 nonclassical); Val448 (3.86 classical); Glu447 (4.84 classical, 3.87 nonclassical); Ser643 (4.87 classical); Ser403 (4.06 classical); Gln521 (4.06 classical); Thr444 (4.02 classical, 3.03 classical, 3.78 nonclassical); Asn464 (4.65 classical); Gly640 (3.90 nonclassical)	Tyr446 (4.62 pi-pi T-shape, 5.07 pi-pi T-shape, 4.62 pi-alkyl)	NA	NA
Rutin	Ser643 (4.11 classical); Thr444 (3.75)	Tyr444 (4.66 pi-pi T-shape); Arg445 (8.57 amid-pi stacked, 9.08 amid-pi stacked)	NA	NA

(C) Molecular dockings performed using the Schrödinger software, and subsequently, the interactions between ligands and residues studied.				
Ligand name	Hydrogen bond (distance Å, subtype)	Hydrophobic interaction (distance Å, subtype)	Electrostatic (distance Å, subtype)	Miscellaneous (distance Å, subtype)

Kaempferol 3-rutinoside-7-sophoroside	Glu262 (3.25 classical, 4.57 nonclassical); Asn260 (4.02 classical); Met375 (4.37 classical, 4.53 classical); Glu263 (4.44 classical, 4.94 classical); Lys285 (4.97 classical); Lys280 (4.13 nonclassical)	NA	NA	NA
Rutin	Asn260 (4.73 classical); Glu263 (4.71 classical, 4.59 classical); Pro258 (3.52 classical); Lys280 (4.59 classical); Lys285 (4.94 classical, 4.72 nonclassical); Gln266 (4.90 classical)	Pro258 (4.78 alkyl, 4.24 alkyl); Tyr380 (5.31 pi-alkyl)	NA	NA

SauPBP2a, *Staphylococcus aureus* penicillin-binding protein 2a.

**Table 3:** Schrödinger Maestro docking score (kcal/mol) of top-ranked flavonoids based on the AutoDock tool, against SauPBP2a active site (PDB ID: 1MWT; chain B).

Compound name	G score	Dock score	H-bond score
Kaempferol 3-rutinoside-7-sophoroside	−12.7	−12.7	−8.2
Rutin	−9.3	−9.3	−2.6
Amentoflavone	−6.3	−6.3	−2.9
Quercetin	−5.9	−5.9	−2.4

SauPBP2a, *Staphylococcus aureus* penicillin-binding protein 2a.

**Table 4:** The relative binding-free energies (kcal/mol) obtained by prime MM-GBSA.

Compound name	MM-GBSA-dG binding energy	MM-GBSA-dG binding, coulomb	MM-GBSA-dG bind (NS)	MM-GBSA-dG bind (NS), coulomb
Kaempferol 3-rutinoside-7-sophoroside	−19.6	−57.4	−43.2	−60.4
Rutin	−27.9	−32.6	−41.6	−27.9
Amentoflavone	−28.9	−32.9	−36.0	−29.5
Quercetin	267.9	−57.9	−29.5	−35.8

MM-GBSA dG bind = complex–receptor–ligand; MM-GBSA dG bind (NS) = complex−receptor (from optimized complex)−ligand (from optimized complex) = MM-GBSA dG bind−receptor strain−ligand strain. NS in the table is the binding energy without considering for the receptor and ligand conformational changes needed for the formation of complex.

## Data Availability

The datasets used and/or analyzed during the current study are available from the corresponding author upon request.

## Abbreviations

*S. aureus*: *Staphylococcus aureus*
MRSA: Methicillin-resistant *Staphylococcus aureus*
PBP2a: Penicillin-binding protein 2a
SauPBP2a: PBP2a in *S. aureus*
*E. coli*: *Escherichia coli*
*P. aeruginosa*: *Pseudomonas aeruginosa*
PDB: Protein Data Bank
EM: Energy minimizing
SDF: Structure data file
RMSD: Root mean square deviation
MD: Molecular dynamics
nM: Nanomolar
MIC: Minimum inhibitory concentration
AgNPs: Silver nanoparticles
MDR: Multidrug-resistant
*V. sphaerocephala*: *Verbesina sphaerocephala*
*K*i: Inhibition constant.

## Supplementary Materials

Supplementary Materials Supplementary Table 1: estimated free binding energy and inhibition constant values between 46 flavonoids, control inhibitors, and the SauPBP2a active site (PBD ID: 1MWT; chain B) achieved from the AutoDock software.
