# Supplementary material for: Methicillin-Resistant Staphylococcus aureus: Docking-Based Virtual Screening and Molecular Dynamics Simulations to Identify Potential Penicillin-Binding Protein 2a Inhibitors from Natural Flavonoids
Source: Int J Microbiol. 2022 May 4;2022:9130700. doi: 10.1155/2022/9130700 (PMC9095385; doi:10.1155/2022/9130700)
Supplement: Supplementary Materials — Supplementary Table 1: estimated free binding energy and inhibition constant values between 46 flavonoids, control inhibitors, and the SauPBP2a active site (PBD ID: 1MWT; chain B) achieved from the AutoDock software. [file 9130700.f1.docx]

**Supplementary Table 1.** Estimated free binding energy and inhibition constant values between 46 flavonoids, control inhibitors, and the SauPBP2a active site (PBD ID: 1MWT; chain B) were achieved from the AutoDock software.

| **PubChem ID** | **Ligand name** | **Binding energy (kcal/mol)** | ***K*i** |
| --- | --- | --- | --- |
| 44258853 | Kaempferol 3-rutinoside-7-sophoroside | -12.0 | 1.4 nM |
| 5280805 | Rutin | -11.1 | 6.8 nM |
| 5281600 | Amentoflavone | -10.2 | 31.6 nM |
| 5280343 | Quercetin | -10.1 | 36.6 nM |
| 44258844 | Kaempferol 3-rutinoside-4'-glucoside | -8.5 | 562.5 nM |
| 5280704 | Apigenin-7-glucoside | -8.4 | 659.6 nM |
| 5280804 | Isoquercitrin | -8.4 | 678.6 nM |
| 5281675 | Orientin | -8.3 | 842.4 nM |
| 5318767 | Nicotiflorin | -8.3 | 847.7 nM |
| 442664 | Vicenin-2 | -8.1 | 1.1 uM |
| 72936 | Sophoraflavanone G | -7.9 | 1.7 uM |
| 9911508 | Astragarin | -7.8 | 1.9 uM |
| 5318998 | Licochalcone A | -7.7 | 2.2 uM |
| 5353915 | Quercetin-3-rhamnoside | -7.6 | 2.6 uM |
| 5280445 | Luteolin | -7.5 | 3.1 uM |
| 5280637 | Cynaroside | -7.5 | 3.2 uM |
| 5280441 | Vitexin | -7.3 | 4.2 uM |
| 5281672 | Myricetin | -7.2 | 5.1 uM |
| 5280459 | Quercitrin | -7.1 | 5.8 uM |
| 14309735 | Xanthogalenol | -7.0 | 7.3 uM |
| 639665 | XanthohuMol | -6.9 | 4.8 uM |
| 638278 | Isoliquiritigenin | -6.9 | 9.0 uM |
| 5281612 | Diosmetin | -6.8 | 9.9 uM |
| 1203 | Epicatechin | - 6.8 | 10.0 uM |
| 5317435 | Fustin | -6.8 | 11.4 uM |
| 5280443 | Apigenin | -6.7 | 11.6 uM |
| 10095180 | Kaempferol 7-O-glucoside | -6.6 | 345.5 uM |
| 5281607 | Chrysin | -6.6 | 13.9 uM |
| 5281654 | Isorhamnetin | -6.6 | 14.4 uM |
| 5280681 | 3-O-Methylquercetin | -6.6 | 14.7 uM |
| 471 | Dihydroquercetin | -6.5 | 18.3 uM |
| 443639 | Epiafzelechin | -6.4 | 18.9 uM |
| 124052 | Glabridin | -6.4 | 19.5 uM |
| 5316673 | Afzelin | -6.4 | 21.0 uM |
| 72281 | Hesperetin | -6.4 | 21.9 uM |
| 5280544 | Herbacetin | -6.4 | 22.1 uM |
| 5282102 | Astragalin | -6.3 | 23.6 uM |
| 629440 | Hemileiocarpin | -6.3 | 24.9 uM |
| 5281670 | Morin | -6.2 | 30.1 uM |
| 25201019 | Ponciretin | -6.2 | 31.3 uM |
| 5280863 | Kaempferol | -6.1 | 32.9 uM |
| 9064 | Catechin | -6.0 | 41.2 uM |
| 10680 | Flavone | -5.9 | 46.6 uM |
| 5281614 | Fisetin | -5.6 | 77.2 uM |
| 439533 | Taxifolin | -5.2 | 2.6 uM |
| 5280378 | Formononetin | -5.2 | 149.7 uM |
| 5289182 | PNM (Penicillin G) (Ctrl **-**) | -6.4 | 22.1 uM |
| 6087 | Methicillin (Ctrl **-**) | -5.5 | 91.2 uM |
| NA | Oxadiazole (Ctrl **+**) | -7.9 | 1.7 uM |
| 137349935 | Ceftobiprole (Ctrl **+**) | -8.8 | 362.2 nM |

*K*i, inhibition constant; SauPBP2a, *Staphylococcus aureus* penicillin-binding protein 2a.
